# Appropriate magnetic resonance imaging techniques for gross tumor volume delineation in external beam radiation therapy of locally advanced cervical cancer

**DOI:** 10.18632/oncotarget.24071

**Published:** 2018-01-06

**Authors:** Yingqiu Song, Beth Erickson, Xiaojian Chen, Guiling Li, Gang Wu, Eric Paulson, Paul Knechtges, X. Allen Li

**Affiliations:** ^1^ Radiation Oncology, Medical College of Wisconsin, Milwaukee, WI, USA; ^2^ Cancer Center, Union Hospital, Tongji Medical College, Huazhong University of Science and Technology, Wuhan, China; ^3^ Radiology, Medical College of Wisconsin, Milwaukee, WI, USA

**Keywords:** cervical cancer, MRI for radiation planning, gross tumor volume delineation, external beam radiation therapy

## Abstract

**Background:**

Accurate delineation of the gross tumor volumes (GTV) is a prerequisite for precise radiotherapy planning and delivery. Different MRI sequences have different advantages and limitations in their ability to discriminate primary cervical tumor from normal tissue. The purpose of this work is to determine appropriate MRI techniques for GTV delineation for external-beam radiation therapy of locally advanced cervical cancer (LACC).

**Materials and Methods:**

GTVs were delineated on the MRI, CT, and PET images acquired for 23 LACC patients in treatment positions to obtain GTVs on CT (GTV-CT), on various MRI sequences including T1 (GTV-T1), T2 (GTV-T2), T1 with fat suppression and contrast (GTV-T1F+), DWI-ADC (GTV-ADC) and on PET were generated using the threshold of 40% of maximum SUV (GTV-SUV40%) as well as SUV of 2.5 (GTV-SUV2.5). MRI, CT and PET were registered for comparison. The GTVs defined by MRI were compared using the overlap ratio (OR) and relative volume ratio (RVR). The union of GTV-T2 and GTV-ADC was generated to represent the MRI-based GTV (GTV-MRI).

**Results:**

The differences between GTV-T2 and other MRI GTVs are significant (*P* < 0.05). The average ORs for GTV-T1, GTV-T1F+, and GTV-ADC related to GTV-T2 were 86.3%, 81.6%, and 61.6% with the corresponding average RVRs 113.8%, 112.3% and 77.2%, respectively. There is no significant difference between GTV-T1 and GTV-T1F+. GTV-ADC was generally smaller than GTV-T2, however, encompassed suspicious regions that are uncovered in GTV-T2 (up to 16% of GTV-T2) because of different imaging mechanisms. There was significant difference between GTV-MRI, GTV-SUV2.5, GTV-SUV40%, and GTV-CT. On average, GTV-MRI is 18.4% smaller than GTV-CT.

**Conclusions:**

MRI provides improved visualization of disease over CT or PET for cervical cancer. The GTV from the union of GTV-T2 and GTV-ADC provides a reasonable GTV including tumor region defined anatomically and functionally with MRI and substantially reduces the conventional GTV defined on CT.

## INTRODUCTION

Cervical cancer is the fourth most common gynecologic malignancy in females worldwide [1, 2] with 85% occurring in developing countries where it is a leading cause of cancer death [3]. Concomitant external-beam radiotherapy (EBRT) and chemotherapy followed by brachytherapy is the recommended treatment strategy for patients with locally advanced cervical cancer (LACC) from IB2 to IVA (Federation of Gynecology and Obstetrics (FIGO) stage). According to clinical guidelines for conformal RT treatment, the target volume of EBRT should include the gross tumor volume (GTV) together with the entire uterine (cervix and body), parametrium and the nodal volumes at risk [4, 5]. Despite the effective control of disease with conventional conformal RT, the large treatment volume can cause serious toxicity later, notably in bowels, vagina and bladder, significantly affecting the quality of life of patient [6, 7].

To reduce toxicity, advanced EBRT techniques, e.g., intensity-modulated radiotherapy (IMRT) and image-guided adaptive radiotherapy (IG-ART), have been introduced to replace conventional conformal RT. Several studies have shown that IMRT decreases treatment-related toxicity while increasing or maintaining disease control [8–10]. In order to reduce the effect of interfraction motion induced by bladder volume variation, several IG-ART strategies have been investigated [4, 11, 12] including a practical approach that selects the plan-of-the-day from a plan library [13, 14]. Due to the high dose gradient offered by IMRT and IG-ART, accurate delineation of the GTV, clinical tumor volume (CTV) and organs at risk (OAR) is a prerequisite for IMRT planning and delivery. Currently, CT-based treatment planning is considered the standard of care for EBRT of LACC. However, as the boundary between tumor and adjacent normal tissue is poorly defined on CT, the GTV is often difficult to be accurately delineated on CT. Fluorodeoxyglucose (FDG) positron emission tomography (PET), a functional imaging technique, has been used to evaluate the metabolic activity of LACC and to define the GTV for LACC [15–17]. A previous study showed that the GTV on PET was significantly different from that defined by CT in 56% of cases [18]. However, PET suffers from poor spatial anatomic resolution and lack of anatomic information. With superior soft-tissue resolution, magnetic resonance imaging (MRI) has a pivotal role in defining the GTV and OAR, especially for determining parametrial and vaginal involvement in patients with an advanced tumor [19–21]. MRI protocols usually include a series of MRI sequences designed to optimally assess a particular region of the body and/or pathological process. In general, T2-weighted and T1-weighted images are most commonly used for anatomy delineation along with other physiological or functional MRI sequences, such as dynamic contrast-enhanced (DCE) MRI [22] and diffusion-weighted imaging (DWI) [23, 24]. DCE-MRI has been valuable in assessing the microvascular structure and functional environment of tumors [25]. DWI and DWI-generated maps of apparent diffusion coefficient (ADC) may provide information about tumor cellularity and aggressiveness. The use of various MRI sequences for GTV delineation of LACC has been reported, particularly for brachytherapy [19]. Differences in the GTVs from different MRI sequences and/or different MR field strengths (1.5T vs. 3T) can be substantial [26, 27].

The choice of appropriate MRI sequence(s) for GTV delineation, particularly for EBRT of LACC, is so far inconclusive. The purpose of this work is to compare the GTV delineations of LACC between CT, PET and various commonly-used 3T MRI sequences and to explore appropriate MRI sequence(s) for the GTV delineation for LACC. The MRI, CT and PET data acquired for EBRT planning for LACC patients are analyzed.

## RESULTS

**Figure 1:**
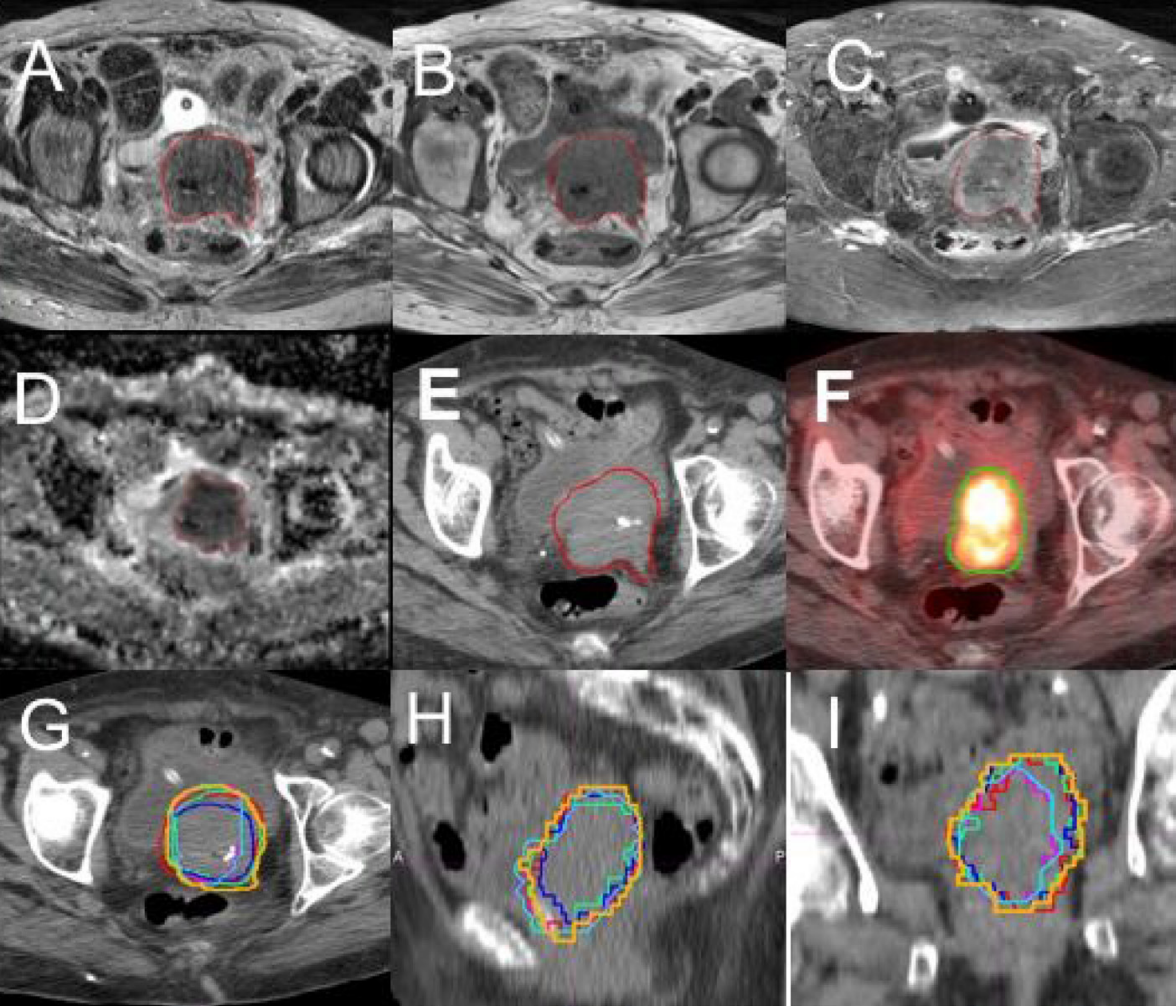
Contours of gross tumor volume (GTV) on multiple imaging modalities for a representative patient (**A**) T2-weighted MRI; (**B**) T1-weighted MRI; (**C**) T1 MRI with fat suppression and contrast (T1F+); (**D**) apparent diffusion-coefficient (ADC) maps; (**E**) CT; (**F**) FDG-PET with SUV = 2.5 threshold. The overlay of the six GTV contours, GTV-T1 (green), GTV-T2 (blue), GTV-T1F+ (red), GTV-ADC (light blue), GTV-SUV_2.5_ (pink) and GTV-CT (brown), on an axial (**G**), a coronal (**H**) and a sagittal (**I**) CT slices are shown.

Differences between the GTVs delineated from the 4 MRI sequences, CT and PET were generally observed. Figure 1 presents GTV contours on the four MRI sequences, CT and PET on a same/similar axial slice for a representative case. It is clear that the GTVs from different imaging modalities or different MRI sequences are quite different. More quantitative data are described below.

### GTV delineation on MRI

**Figure 2:**
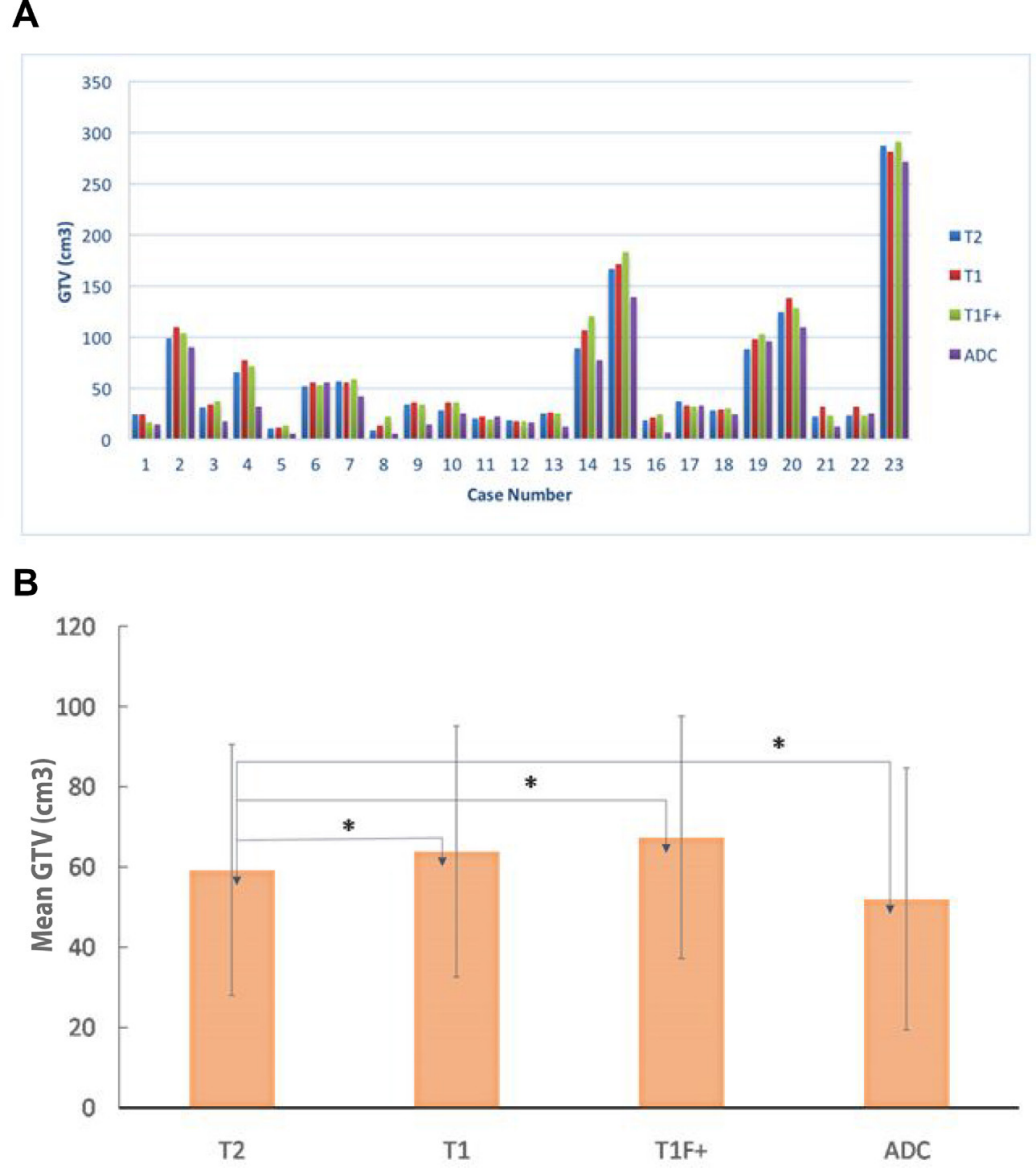
(**A**) a comparison of gross tumor volumes (GTVs) obtained from four MRI sequences T1, T2, T1 with fat suppression and contrast (T1F+), and apparent diffusion-coefficient (ADC) for all the 23 patients studied; (**B**) the mean and SD of the GTVs from T2, T1, T1F+, and ADC for all the patients studied. “^*^” indicates statistically significant differences between the GTVs.

**Figure 3:**
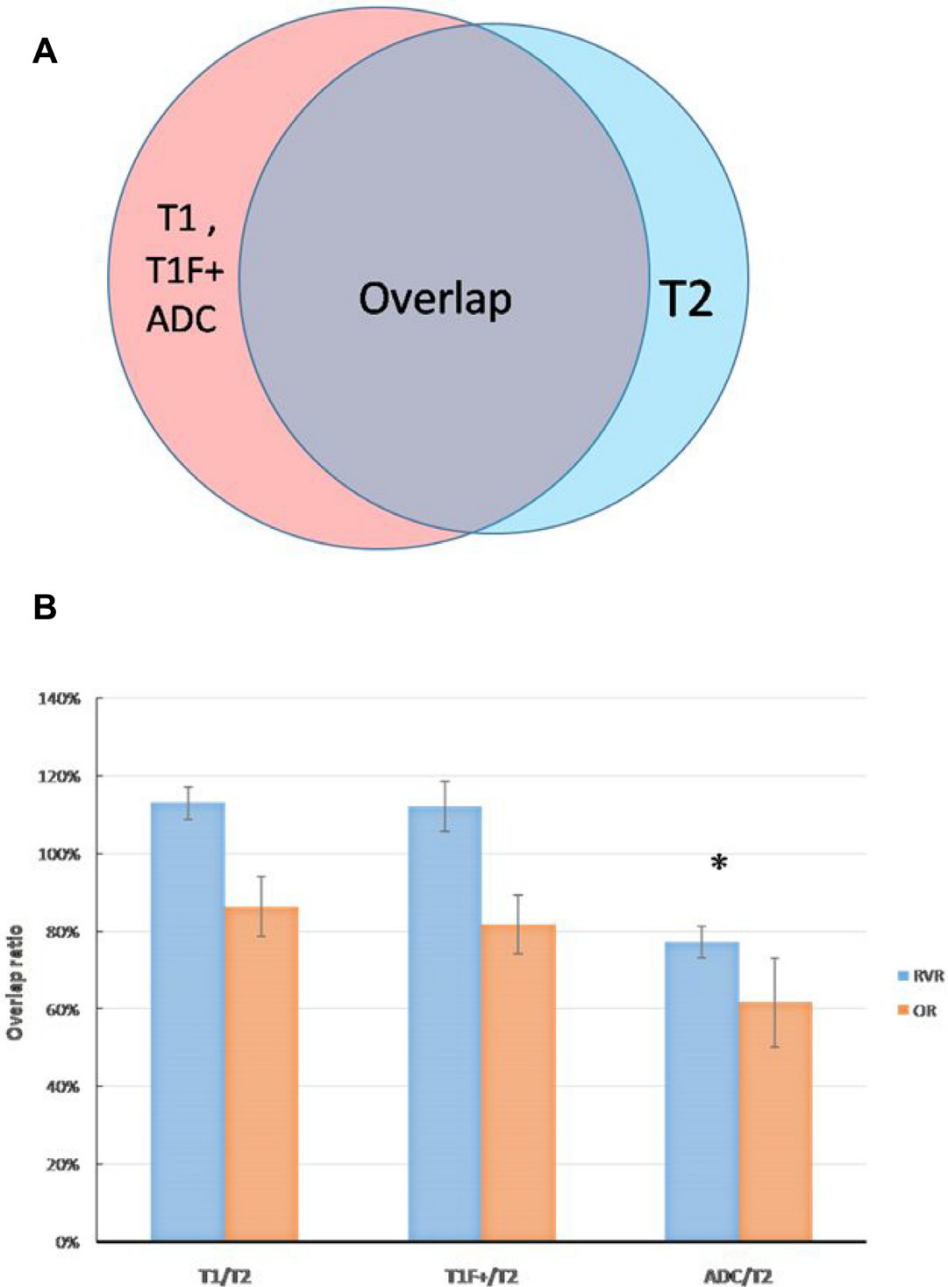
(**A**) a sketch to determine the relative volume ratio (RVR) and the overlap ratio (OR) for GTV-T1, GTV-T1F+ and GTV-ADC with respective to GTV-T2, RVRs representing the relative concordance between GTV-T2 and GTV-T1, GTV-T1F+, GTV-ADC, and ORs showing the percentage of overlap between the volumes. (**B**) the mean and SD of the RVRs and ORs of GTV-T1 and GTV-T2, GTV-T1F+ and GTV-T2, GTV-ADC and GTV-T2. “^*^” indicates statistically significant differences.

The volumes of GTV-T1, GTV-T2, GTV-ADC, and GTV-T1F+ for each of the 23 patients are compared in Figure 2A. The average GTV- T1, GTV-T2, GTV-ADC and GTV-T1F+ over all the patients are 62.8 ± 62.1, 58.4 ± 61.2, 50.9 ± 59.8, and 66.4 ± 64.9 cm^3^, respectively. The differences in volume between GTV-T2 and the other three MRI-based GTVs are significant (*P* < 0.05) (Figure 2B). The average ORs for GTV-T1, GTV-T1F+, and GTV-ADC relative to GTV-T2 were 86.3%, 81.6%, and 61.6% while the corresponding average RVRs were 113.8%, 112.3%, and 77.2%, respectively. These data are displayed in Figure 3A and 3B where the significant differences are indicated by asterisks. There is no significant statistical difference between GTV-T1 and GTV-T1F+(*P* > 0.05).

**Figure 4:**
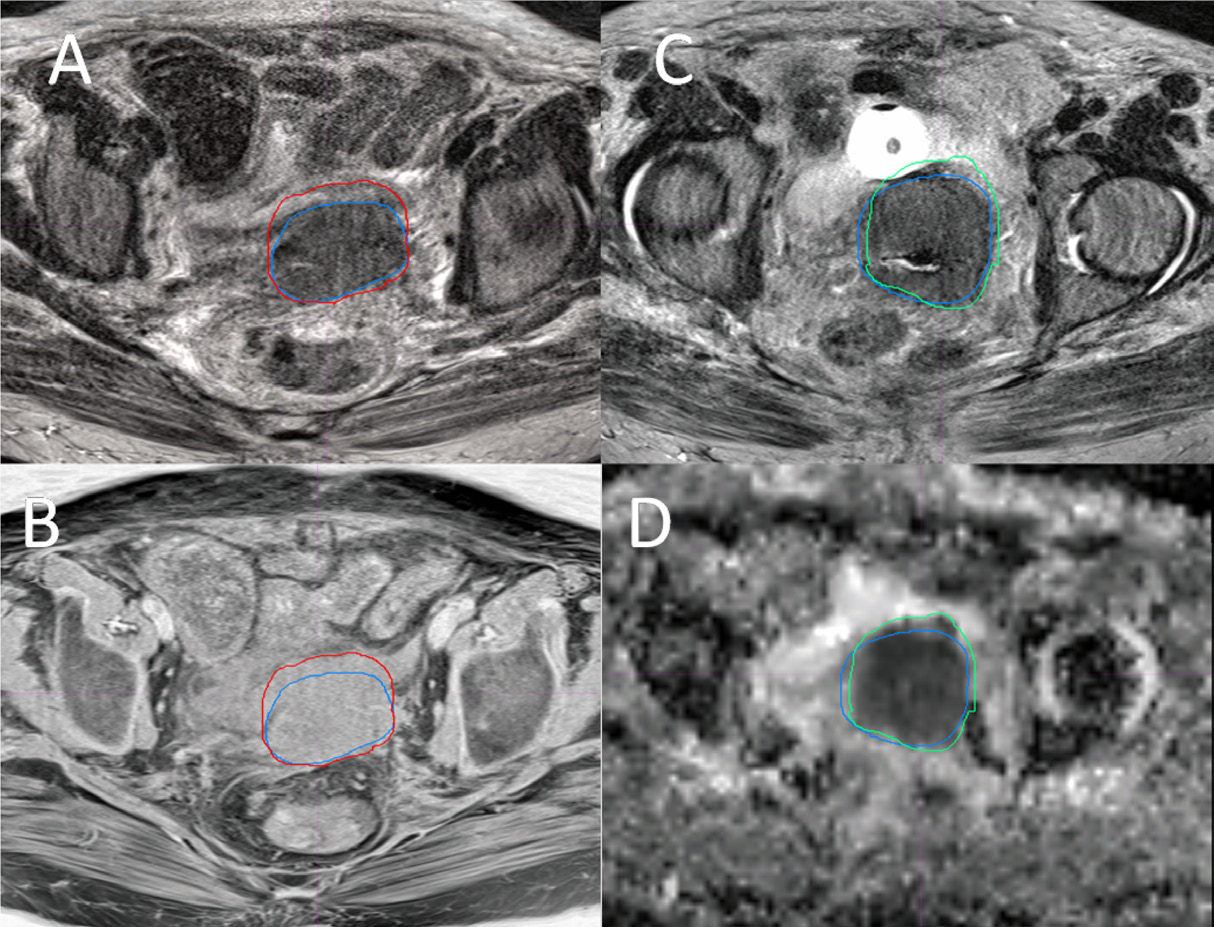
Overlay of the GTVs from four MRI sequences of a Stage IVA cervical tumor Overlay of GTV-T2 (blue) and GTV-T1 (red) on a T2 (**A**) and a T1 (**B**) slices, and overlay of GTV-T2 (blue) and GTV-ADC (green) on a T2 (**C**) and an ADC (**D**) slices, where GTV-T2 = 98.4 cm^3^, GTV-T1= 110.2 cm^3^, and GTV-ADC = 90.8 cm^3^.

Figure 4A and 4B show the overlay of GTV-T1 and GTV-T2 for a representative case. As it is shown, GTV-T1 has a larger volume than GTV-T2, which is generally true for other cases. The difference is mainly due to the lower soft tissue contrast at the tumor boundary in T1 compared to T2. On the contrary, Figure 4C and 4D show that GTV-ADC is generally smaller than GTV-T2, however, encompassing suspicious regions that are sometimes not revealed by T2 because of different imaging mechanisms. The uncovered volume accounts for up to 16% of the GTV-T2. To resolve the issues with GTV-T2 and GTV-ADC, we propose to create a GTV based on the union of the GTV-T2 and GTV-ADC, referred to the MRI-based GTV (GTV-MRI).

### Comparison of MRI-based with CT and PET based GTV delineations

**Figure 5:**
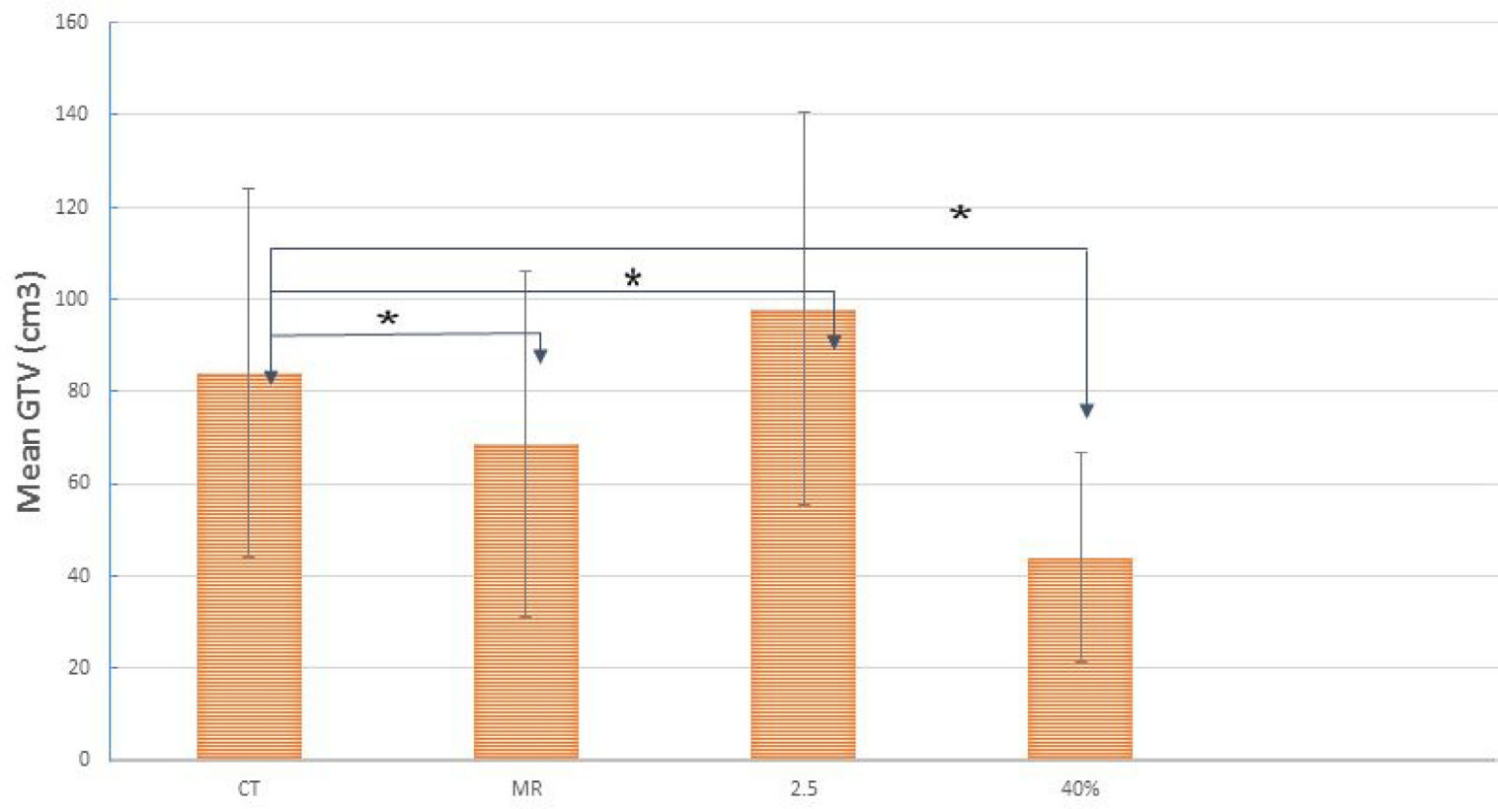
The mean and SD of the GTVs from CT, GTV-MRI (the union of GTV-T2 and GTV-ADC), GTV-SUV_2.5_ and GTV-SUV_40%_ for all the patients studied “^*^” indicates statistically significant differences.

Figure 5 shows the overlap of GTV-MRI GTV-CT, GTV-SUV_2.5_ and GTV-SUV_40%_. The mean values of GTV-CT, GTV-MRI, GTV-SUV_2.5_ and GTV-SUV_40%_ were 83.98 ± 79.7, 68.52 ± 61.2, 98.32 ± 84.8 and 44.62 ± 45.6 cm^3^, respectively. There were significant differences between these GTV contours (*P* < 0.05). On average, GTV-MRI is 18.4% smaller than GTV-CT for the cases studied.

## DISCUSSION

Due to its high soft tissue contrast and ability to distinguish border between tumors and normal tissues, MRI has been recommended for diagnosis, staging, treatment planning and treatment prognosis evaluation of cervical cancer by the GYN GEC-ESTRO working group [4, 28]. It has been reported that the use of MRI in treatment planning can provide clearly defined target volumes and can be helpful to evaluate prognosis for cervical cancer [29, 30]. In particular, the T2-weighted MRI can improve target and OAR definition in brachytherapy [4, 28, 29].

Different MRI sequences have different advantages as well as limitations in their ability to discriminate primary cervical tumor from normal tissue. In general, T2-weighted and T1-weighted MRIs are considered the choice of sequences for gross anatomic structures. However, T1-weighted images can sometimes show blurred tumor borders because of the equivalent T1 signal between cervical tumor and normal tissue [31], implying that the T1 MRI may not be able to exactly reveal the extension of tumor invasion. The cervical tumor displays uniform or non-uniform middle to high signals on T2W TSE images providing better contrast between normal basal cervical tissue (low signal) and parametrial tissue (high signal). For this reason, the T2-weighted is often used to define the GTV of cervix [31]. T1 with fat suppression and contrast (T1F+) may enhance the signal of tumor. However, the signal can still be higher, lower or equal to that from the normal cervix [32]. Studies investigating the efficacy of T2-weighted, dynamic, and post contrast T1-weighted images to assess the degree of stromal invasion and parametrial involvement by cervical carcinoma include some controversy [33, 34]. Tsuda et al. showed that T2-weighted images permitted the most accurate evaluation of stromal invasion by uterine tumors [33]. Over diagnosis due to abnormal intensity of cervical stroma was observed more frequently on dynamic and contrast T1-weighted images than on T2-weighted images [34]. ADC-DWI, measuring the density of tumor cells and diffusion coefficient of water molecules can quantitatively evaluate the invasion of tumor [35]. From an ADC map, it is easy to distinguish the tumor from normal cervical tissue due to the inherent low signal of cervical tumor [36].

In this study, differences were shown in the GTVs defined with different MRI sequences. In general, GTV-ADC is the smallest and GTV-T1 and GTV-T1 F+ are the largest, with GTV-T2 in between. The average ORs for GTV-T1, GTV-T1F+, and GTV-ADC relative to GTV-T2 were 86.3%, 81.6%, and 61.6% while the corresponding average RVRs were 113.8%, 112.3% and 77.2%, respectively. As shown in Figure 4A and 4B, GTV-T1 is generally larger than GTV-T2 mainly due to the lower soft tissue contrast at the tumor boundary in T1 images than T2 images. It is seen that the tumor border is clear, shown as the blue contour on T2 (Figure 4A), but not on T1 (Figure 4B)) (stage IVA, GTV-T2 98.4 cc, GTV-T1 110.2 cc). The front border of the GTV-T1 encloses a part of normal tissue (the small bowel wall). In contrast, as shown in Figure 4C and 4D, GTV-ADC was generally smaller than GTV-T2 and encompassed suspicious regions that are sometimes not indicated by T2 because of different imaging mechanisms. The uncovered volume accounts for up to 16% of the GTV-T2. Due to these reasons, the GTV-MRI, generated from the union of the GTV-T2 and GTV-ADC, may be more appropriate than individual GTV-T2 or GTV-ADC.

To validate the use of MRI for GTV delineation, a few studies have compared the tumor volumes from various imaging modalities with the pathological tumor volume from surgical specimens [37, 38]. Van de Schoot et al. [37] compared the pathological tumor volumes after surgery with the GTVs defined by T2 images before the surgery for early staging cervical cancer and reported that GTV-T2 underestimated the tumor volume. They suggested a 12-mm margin should be extended from GTV-T2 in order to cover 95% volume of the tumor. On the other hand, Zhang et al. [38] compared the GTVs defined by various images (CT, T2, and PET) to the GTV defined by histologic exam of the surgical specimen. They showed that the maximum diameter of T2-GTV is larger than the GTV defined by the surgical specimen while the GTV with FDG-PET 40% SUVmax is closest to the GTV of pathology. In ADC images, while its anatomic resolution is low, the contrast between tumor and normal tissue is much higher than that in T1 and T2 images due to its quenching the background [39]. These drawbacks, T2 and ADC, can be compensated, at least partially, by combining ADC with T2 images. A recent study by a GEC ESTRO group investigating ADC-values for different tumor tissue characteristics also suggested that combined T2 and ADC-DWI MRI might be better than T2 imaging alone in the defining of tumor target [36].

In our study, comparison of various GTVs from MRI, CT, and PET allows a direct inspection of the advantages and disadvantages of these different image modalities for LACC GTV definition. There were significant differences between GTV-MRI, GTV-SUV_2.5_, GTV-SUV_40%_, and GTV-CT. On average, GTV-MRI is 18.4% smaller than GTV-CT. High soft tissue contrast with MRI reduces GTV delineation uncertainty as compared to CT and PET. Combing MRI and FDG-PET may further improve the tumor definition. Generally speaking, the improved GTV delineation with using planning images of good soft-tissue contrast and functional information (such as MRI) increases target coverage and/or normal structure sparing. If such images are used to guide EBRT delivery (e.g., using MR-Linac), the accurate GTV delineation will also improve overall performance of adaptive RT. Furthermore, the reduced GTV as a result of the accurate GTV delineation based on MRI can lead to reduced radiation fields in EBRT. The small radiation fields reduce the irradiation of the adjacent normal tissues. Radiation induced normal tissue injury has been a major factor limiting the effectiveness of EBRT for cervical cancer. In addition, with improved normal tissue sparing, the radiation dose can be safely escalated if it is necessary.

There are several limitations in this work. First, the sample size was small. A large sample will be needed to further confirm statistical findings. Second, as one of the important imaging parameters, different b values show different ADC values (b = 0, 500, 1000 are selected in our research). A higher b value is helpful to reduce the penetration effect of T2 and improve the accuracy of GTV-ADC by obtaining a stable ADC-DWI image. Third, the rigid-body image registration used may introduce registration errors. There may be non-rigid organ motion (e.g., deformation) between the image acquisitions. Deformable image registration (DIR) might help, but accurate DIR tools are generally lacking for the fusion of CT, MRI and PET.

MRI provides improved visualization of disease over CT or PET for cervical cancer. However, different MRI sequences can lead to different GTV delineations. Among the sequences studied, the union of GTV-T2 and GTV-ADC represents a reasonable GTV definition for MRI (GTV-MRI), which includes the most possible disease region but least excessive surrounding normal tissue. The GTV-MRI is substantially smaller than conventional CT defined GTV-CT and may be used for precise IMRT planning. Further studies are required to confirm that the use of GTV-MRI for EBRT of cervical cancer is appropriate.

## MATERIALS AND METHODS

### Patient data

MRI, CT and PET data acquired for 23 LACC patients with a median age of 56-years were retrospectively analyzed in this IRB approved study. All patients had a histological diagnosis of cervical carcinoma and were staged IB2-IVA (locally advanced disease) according to the International Federation of Gynecology and Obstetrics (FIGO) classification using standard pretreatment workup. All patients were treated with EBRT of 50 Gy in 25 fractions or 50.4 Gy in 28 fractions followed by brachytherapy of 28 Gy in 4 fractions as a boost to the cervix and primary tumor. Patient characteristics are listed in Table 1.

**Table 1:** Clinical characteristics of the 23 patients studied

Characteristics	Number of cases (%)
Age (y), median (range)	56 (30–83)
Stage(FIGO)	
IB2	6 (26.1)
IIB	8 (34.8)
IIIA IIIB	2 (8.7) 4 (17.4)
IVA	3 (13.0)
Lymph node involvement	
none	15 (65.2)
yes	8 (34.8)
Pathologic type and histology grade	
Squamous cell carcinoma High grade differentiated(G1) Moderate grade differentiated(G2) Poor grade differentiated(G3)	19 (82.6) 4 (21.1) 5 (26.3) 10 (52.3)
Adenocarcinoma	3 (13.0)
Adenosquamous carcinoma	1 (4.3)
Time interval from CT to MRI scanning (days), median(range)	1 (0–20)

Abbreviation: FIGO :International Federation Gynecology and Obsteterics

### Image acquisition

#### MRI acquisition

The MRI data were acquired on a 3T, 70-cm bore MR scanner (Vero, Siemens, Erlangen, Germany) equipped with a body radiofrequency (RF) coil, a spine phased-array RF coil and two flexible phased-array coils. All patients were set up in the same treatment position as in the CT and PET scans. The MR protocol consisted of fast-recovery fast spin-echo T2 (T2-FRFSE; T2), three-dimensional (3D) T1 without fat suppression (T1) and apparent diffusion-coefficient (ADC) maps obtained from diffusion-weighted imaging (DWI). A 2D single-shot, twice refocused spin-echo, echo-planar imaging was used for DWI acquisition. The contrast-enhanced MRI was performed using fat-suppressed T1 weighted gradient-echo sequence (T1C+) with 20 ml of intravenous MultiHance (Barcoo diagnostics, Monroe Township, NJ) injected at 3ml/s. The average total acquisition time of this protocol was approximately 30–40 minutes.

The technical parameters were as follows: T2FRFSE: time to repetition (TR) / time to echo (TE), 3600/85 ms, voxel size 0.68 × 0.68 × 5 mm, dimensions 320 × 320 × 36; thickness 5 mm;T1-Weight: TR/TE, 600/11 ms, voxel size 0.8 × 0.8 × 5 mm, dimensions 384x384x38; thickness 5 mm; Axial -2D echo-planar (EP) DWI-ADC: TR/TE,13200/80 ms, voxel size 2.0×2.0×5 mm, dimensions 128 × 128 × 50; thickness 5 mm; b = 0,500,1000. AX T1 (F)+C: TR/TE, 600/11 ms, voxel size 0.6 × 0.6 × 5 mm, dimensions 384 × 384 × 38; thicker 5 mm.

### CT and PET/CT acquisition

The planning CT was acquired using a large bore CT (HighSpeed, GE) with 120 kVp, auto-mAs (range: 182–285), 2.5 mm slice thickness, and < 1 mm pixel size. Free breathing PET/CT images were performed using a PET/CT system (Discover Loadstone; GE). Patients were asked to fast for a minimum of six hours before acquisition of the PET. A blood glucose level was checked just before the administration of ^18^F-FDG to exclude patients with hyperglycemia. Approximately 16 ± 3.5 mCi (range: 12–20.5 mCi) of ^18^F-FDG was injected intravenously. Sixty minutes later, PET images were obtained using a whole body protocol. A registered non-contrast enhanced axial CT scan was obtained through the same levels to use for attenuation correction and reviewed to localized FDG uptake. All PET images were reconstructed with ordered-subset expectation maximization. The slice thickness was 4.25 mm and the pixel spacing was 3.91 × 3.91 mm^2^. All MRI, CT and PET images were acquired in the treatment position using the same immobilization device on flat tabletop.

### GTV delineation

Image registration and contour delineation of MRI, CT and PET/CT data were performed using an imaging processing software tool (MIM Software Inc., Cleveland, OH). The MRI sequences were all intrinsically registered automatically. The registrations between MRI, CT, PET/CT were first attempted with rigid-body method and then manually adjusted based on local matching of soft tissues. The GTVs on CT and four sequences of MRI (T2-FSFSE, T1-weighted, DWI-ADC and T1 with fat suppression and contrast (T1F+), referred as GTV-CT, GTV-T2, GTV-T1, GTV-ADC and GTV-T1F+ were manually delineated by a radiation oncologist with more than 10 years of experience in radiotherapy for cervical cancer and verified by another radiation oncologist with more than 20 years of experience in radiotherapy for cervical cancer based on the guidelines from the European Society for Therapeutic Radiology and Oncology (GEC-ESTRO) and considering the other earlier general instructions reported for MR-based delineation [40]. The GTV contours were generated on the axial slices while referring to the sagittal and coronal images during the delineation. The GTV-T1, GTV-ADC and GTV-T1F+ contours were then populated to the T2 images to compare with the GTV-T2. The differences were measured with the overlap ratio (OR) and relative volume ratio (RVR), where OR was defined as the ratio of the overlap volume between GTV-T2 and GTVs defined by other MRI sequences and RVR was calculated as the ratio of GTVs relative to GTV-T2 (GTV-T1/GTV-T2, GTV-ADC/GTV-T2 and GTV-T1F+/GTV-T2). The union of the GTV-T2 and GTV-ADC was created and referred to as the MRI-based GTV (GTV-MRI).

Based on the fused PET-CT data, GTVs on PET were generated using two different thresholds of SUV, SUV = 2.5 and 40% of SUVmax, referred as GTV-SUV_2.5_ and GTV-SUV_40%_, respectively. For each case, the GTV was delineated first on the MRIs, and then, on the CT and PET. All image sets were blinded and were presented in a random order to the observer. The obtained GTVs contours from MRI, CT, and PET were compared based on rigid-body registration of appropriate images.

### Statistical analysis

All analytical variables in this study were continuous and presented as means and standard deviations (mean ± SD). One-way analysis of variance (ANOVA) with Bonferroni post hoc test was performed to assess differences among different groups. All analyses were performed with the SPSS package (release 22.0). Two-sided hypothesis testing was used for all analysis and a predetermined level of *P* < 0.05 was considered statistically significant.

## Abbreviations

GTV: Gross tumor volumes
EBRT: External-beam radiation therapy
LACC: Locally advanced cervical cancer
MRI: magnetic resonance imaging
FIGO: Federation of Gynecology and Obstetrics
IMRT: Intensity-modulated radiotherapy
IG-ART: Image-guided adaptive radiotherapy
DCE: Dynamic contrast-enhanced
DWI: Diffusion-weighted imaging
ADC: Apparent diffusion coefficient
